# Prevention of Brain Metastases

**DOI:** 10.3389/fneur.2018.00758

**Published:** 2018-09-28

**Authors:** Joseph A. Bovi

**Affiliations:** Department of Radiation Oncology, Medical College of Wisconsin, Milwaukee, WI, United States

**Keywords:** CNS metastases, prevention, SRS, WBRT, immunotherapy, targeted therapy (TT)

## Abstract

The incidence of brain metastases is projected to rise because survival rates of lung cancer, breast cancer, and melanoma continue to improve (1). The brain is being identified as a sanctuary site for harboring metastases despite excellent control of extracranial disease. This is thought to occur because the drug therapies that control extracranial disease have limited central nervous system (CNS) penetration. The development of brain metastases is a devastating diagnosis affecting both quality of life (QOL) and survival. Symptoms after diagnosis can include headache, nausea, vomiting, seizure, neurocognitive decline, and focal neurologic deficit. Some of these symptoms can be irreversible even after successful treatment of intracranial disease. Treatment of brain metastases often necessitates surgery and radiation. There have been some reports of systemic therapies offering an intracranial response however long-term data is lacking. These treatments for CNS metastases can also lead to neurocognitive sequelae impacting quality of life. Therefore, preventing disease from spreading to the brain is a topic that has generated much interest in oncology. Prophylactic cranial Irradiation (PCI) has been used in leukemia, small cell lung cancer (SCLC), and non-small cell lung cancer (NSCLC). While showing effectiveness in preventing intracranial disease development, its carries with it side effects of neurocognitive decline that can affect QOL. There are Clinical trials exploring novel delivery of PCI and concurrent neuroprotective drug therapy to try to mitigate these neurocognitive sequelae. These will be important trials to complete, as PCI has shown promise in controlling disease and prolonging survival in select patient populations. There are also drug therapies that have shown efficacy in preventing CNS metastases development. This review will explore the current therapies available to prevent CNS metastases.

## Standard brain mets treatment

Standard therapies for brain metastases often include surgery, whole brain radiotherapy (WBRT), stereotactic radiosurgery (SRS), or a combination of these treatment modalities. The decision for utilizing these therapies are often dependent upon the number of lesions, their location, and the severity of patients' symptoms.

The routine use of WBRT has been challenged with recent publications showing improved cognitive outcomes and equivalent survival in patients treated with SRS compared to SRS and WBRT in patients with limited brain metastases (2). In addition, SRS is also being favored over WBRT following resection of metastases as recent data has also shown good local control and equivalent survival with less neurocognitive decline in patients where WBRT following surgery was omitted (3, 4). Regardless of the reduction in neurocognitive sequelae when WBRT is withheld, it's important to recognize that patients still experienced neurocognitive decline even when focused radiotherapy was administered. This is a fact that is frequently omitted in the discussion of the results of these trials. The mere presence of metastatic disease can lend itself to neurocognitive symptoms. These may not be outwardly apparent to the patient or clinician but in trials where pre-treatment cognitive assessments were performed, pre-WBRT neurocognitive symptoms were uncovered with testing (5, 6). This underscores the need for prevention of brain metastases as opposed treatment after the development of intracranial disease.

## PCI in the management of acute lymphoblastic leukemia (all)

PCI was initially introduced in order to address metastatic disease to the CNS in childhood leukemia. The CNS was known to be a sanctuary site for leukemic cells and CNS relapses were common and carried with it a poor prognosis (7–9). Early studies had shown that patients with high risk features (young age at diagnosis, T cell phenotype, WBC count >50,000–100,000, extra-medullary disease, presence of Philadelphia chromosome, and poor response to induction chemotherapy) had poor survival even after they had achieved remission, and this was attributed to CNS relapses (7–9). In high risk populations, PCI has been shown to decrease the rate of CNS recurrences from 42 to 100% down to 6% (10). These impressive results have been seen in both the pediatric and adult populations (11).

The unfortunate result of delivering radiation therapy to the brain in this disease process is the long-term repercussions of CNS radiation toxicity. Some of the side effects that children developed as a result of these therapies were neurocognitive decline, mood disturbances, short stature, abnormal skull growth, endocrinopathies, and secondary malignancies. As a result of these side effects the radiotherapy dose has been aggressively decreased from 24 to 12 Gy in the hopes of avoiding some or all of these long-term toxicities (12–14).

Currently, leukemia CNS prophylaxis without PCI has been the preferred approach. As an example, the Berlin-Franfurt-Munster (NHL-BFM 95) trial showed that in Stage III–IV lymphoblastic leukemia who received high dose systemic methotrexate, including intrathecal (IT) methotrexate, had very low rates of CNS relapse comparable to historic control who had received PCI (15). Additionally, the Children's Leukemia Group showed that even in patients with CNS involvement at diagnosis had high rates of cure and low rates of CNS relapse with appropriate systemic and IT therapies (16).

Current management of ALL, even with high risk features, excludes PCI. However, the early use of this treatment modality was the initial pioneering effort that led to cures of childhood ALL and paved the way for this treatment modality to be utilized in other malignancies where metastases can be harbored in the CNS and shielded from effective systemic chemotherapies.

## PCI in small cell lung cancer

Small cell lung cancer (SCLC) is an additional malignancy where CNS failure rates are approximated to be 50–60% at 2 years following diagnosis (17). CNS failure in SCLC carries with it a poor prognosis (18). As a result of these high rates of CNS failure, consideration of delivering PCI to improve local CNS control was considered.

Initial early trials did not show a clear benefit to the delivery of PCI in SCLC (19). These early trials did not separate patients into limited disease (LD) or extensive disease (ED) or perform appropriate re-staging for response to chemotherapy prior to the delivery of PCI. The failure to show improvements in survival was likely due to the competing risk of death from systemic disease progression or the presence of CNS disease prior to the delivery of “prophylactic” CNS radiation. What became evident was that patients who had a complete response to systemic chemotherapy in LD SCLC and were re-staged prior to the delivery of PCI benefitted from PCI with both local control and survival (Table 1). The Aurperin meta-analysis demonstrated that the use of PCI at varying dose and fractionation schedules who had a complete response to systemic chemotherapies had a 50% reduction in the development of brain metastases and an improvement in overall survival (20.7% PCI vs. 15% observation) (25). A more recent analysis of 12 trials by Meert et al. showed similar results. PCI decreased brain metastases and improved survival in patients achieving a complete response (CR) after chemotherapy with hazard ratio [HR] of 0.48 (95% CI 0.39–0.60) for incidence of brain metastases, and HR of 0.82 (95% CI 0.71–0.96) for survival. However, when patients with less than a CR to chemotherapy were included in this analysis, the benefit of PCI on survival became non-significant (HR 0.94, 0.87–1.02) (27).

**Table 1:** Randomized trials of PCI in SCLC.

**Trial**	**Years**	**Patients (*n*)**	**PCI dose (Gy# of fractions)**	**Brain metastasis rate (%) (PCI vs. no PCI)**	***p*-value**	**Survival (PCI vs. no PCI)**	***p*-value**	**References**
UMCC	1977–1980	29	30/10	0 vs. 36	0.02			(20)
Okayama	1981–1986	46	40/20	22 vs. 52	< 0.05	Median 21 months vs. 15 months	0.097	(21)
PCI-85	1985–1993	300	24/8	40 vs. 67 (2 year rate)	< 10^−13^	29 vs. 21.5 (2 year)	0.14	(18)
UKCCCR-RORTC	1987–1995	314	Variable	38 vs. 54 (3 year rate)	0.00004	21 vs. 11 (3 year)	0.25	(22)
PCI-88	1988–1994	211	Variable	44 vs. 51 (4 year rate)	0.14	22 vs. 16 (4 year)	0.25	(23)
ECOG-RTOG	1991–1994	32	25/10	24 vs. 53	NS	Median 15.3 months vs. 8.8 months	0.25	(24)
Auperin meta-analysis	1977–1995	987	Variable	33.3 vs. 58.6(3 year rate)	< 0.0001	20.7 vs. 15.3(3 year)	0.01	(25)

*Adapted from Prophylactic cranial irradiation: recent outcomes and innovations (26)*.

Recommendations for PCI in patients with ED-SCLC is less clear. Auperin's meta-analysis included a small number of patients with ED-SCLC and in those patients who achieved a complete response (CR) to systemic chemotherapy there was better survival and lower rates of brain metastases when PCI was administered (25).

In addition to this data, the European Organization for Research and Treatment of Cancer (EORTC) performed a Phase III trial investigating the role of PCI in patients with ED SCLC who had partial response (PR) or CR to chemotherapy (28). The risk of brain metastases at 1-year was significantly reduced in the PCI group (14.6% PCI vs. 40.4% No PCI), and the 1-year survival rate was also superior (27.1% PCI and 13.3% No PCI). A criticism of this study was its lack of re-staging brain MRI in asymptomatic patients which may have led to inclusion of patients who may have harbored brain metastases.

More evidence in support of PCI in ED-SCLC came from a North Central Cancer Treatment Group analysis examining patients with LD and ED-SCLC with stable disease following chemotherapy and thoracic radiotherapy. Three hundred eighteen patients were enrolled, and this showed improvement in survival at 1 and 3 years with limited toxicity using traditional radiation dose fractionation (29).

There are other studies that question the routine use of PCI in ED-SCLC. The Japanese closed their phase III trial early due to the lack of survival seen in patients who received PCI (25 Gy in 10 fractions). Median survival was shown to be 10.1 months in those receiving PCI compared to 15.1 months without PCI (*p* = 0.091). However, there was a significant reduction in the development of brain metastases (32% PCI vs. 58% No PCI) which matches the 50% reduction in brain metastases development seen in patients with LD-SCLC where PCI is administered (30).

There is a clear role for PCI in LD-SCLC who demonstrate a CR to systemic chemotherapy with improvements in both local control and survival. The routine use of PCI in ED-SCLC is less clear. However, it seems very reasonable to consider administering this therapy in patients with ED-SCLC who show response to initial systemic chemotherapies and who have not developed brain metastases upon restaging of the CNS prior to PCI delivery.

## Roll of PCI in non-small cell lung cancer (NSCLC)

Brain metastases occur with frequency in patients diagnosed with NSCLC and are also one of the first sites of relapse. Patient with early stage (I–II) disease are less likely to be diagnosed with brain metastases compared to those with more advanced disease (Stage III) (31–37).

The role of PCI in NSCLC is not as well established as it is in those with SCLC. However, there are some older studies that demonstrated PCI reduced development of CNS metastases and prolonged the time to develop intracranial disease. Cox et al. had shown that PCI decreased the incidence of CNS metastases from 13% to 6% (*p* = 0.038) (38). Umsawasdi et al. showed a decrease in CNS metastases from 27% (No PCI) to 4% (PCI) (*p* = 0.002) with an increase in CNS metastases free survival (39).

However, the biggest criticism of PCI in NSCLC is that, while this treatment modality demonstrates reductions in the development of brain metastases, there is not a corresponding improvement in overall survival. As an example, the RTOG tried to demonstrate a benefit of PCI in Stage II and III NSCLC. With 187 patients enrolled, there were non-significant reductions in the development of brain metastases but also a non-significant reduction in survival in the PCI arm (40). There was however one trial that showed a significant benefit in brain metastases reduction and survival (41).

Based upon these mixed results, the RTOG tried to definitively answer the question of the benefit of PCI in NSCLC with RTOG 0214. This was a Phase III trial with Stage IIIA and IIIB NSCLC. Three hundred fifty-six patients were accrued to this study. After definitive treatment, patients were randomized to PCI, 30 Gy in 15 fractions or observation. This study closed early due to poor accrual. Unfortunately, it failed to show a difference in overall survival between the two arms, however, there was a statistically significant reduction in the development of brain metastases (18.0% No PCI vs. 7.7% PCI, p = 0.004) (42).

Based upon these trials, the routine use of PCI in NSCLC is not routinely recommended. (Table 2).

**Table 2:** Randomized trials evaluating PCI in NSCLC.

**Trial**	**Year of publication**	**Patients (*n*)**	**PCI dose (Gy# of fractions)**	**Brain metastasis rate (%) (PCI vs. no PCI)**	***p*-value**	**Survival (PCI vs. no PCI)**	***p*-value**	**References**
VALG	1981	281	20/10	6 vs. 13	0.038	Median 8.2 months vs. 9.7 months	0.5	(38)
MDACC	1984	97	30/10	4 vs. 27	0.02			(39)
RTOG 8403	1991	187	30/10	9 vs. 19	0.10	Median 8.4 months vs. 8.1 months	NS	(40)
SWOG	1998	254	37.5/15 or 30/10	1 vs. 11	0.003	Median 8 months vs. 11 months	0.004	(41)
RTOG 0214	2011	356	30/15	7.7 vs. 18 (1 year rate)	0.004	75.6 vs. 76.9 (1 year)	0.86	(42)

*Adapted from Prophylactic cranial irradiation: recent outcomes and innovations (26)*.

## Side effects and QOL

Cranial radiation can cause significant neurologic toxicity that can negatively impact QOL. This argument is used for forgoing PCI especially in settings where a survival benefit is not realized. However, when PCI is omitted, the competing risk of neurologic sequelae caused by the emergence of CNS metastases must also be considered (28).

Earlier studies reporting on the neurocognitive impact of PCI were small, retrospective, and did not establish a pre-treatment baseline (43). The absence of a pre-treatment baseline is critical because there are many factors that can lead to neurocognitive decline in patients other than the presence of metastatic disease or radiotherapy. Age, smoking, paraneoplastic syndromes, and depression are just a few factors that can lead to neurocognitive symptoms in the absence of radiotherapy. This is why it is absolutely necessary to perform neurocognitive assessments on patients at baseline to truly measure the impact that radiotherapy can have on posttreatment neurocognition.

Modern series assessing the efficacy of PCI have included more robust and reliable assessments of cognitive function assessed both before and after the administration of radiotherapy like mini mental status exam (MMSE), Hopkins Verbal Learning Test (HVLT) and Controlled Oral Word Association (COWA). Cognitive evaluation of RTOG 0212 showed a correlation between higher-dose PCI and increased, chronic neurological toxicity, but this was not associated with an impact on HVLT score (44).

Pooled analysis of RTOG 0212 and RTOG 0214 reported that patients treated with PCI had a greater risk of self-reported neurocognitive decline at 6 months (odds ratio [OR] 3.60, 95% CI 2.34–6.37; *p* < 0.0001) and 12 months (OR 3.44, 1.84–6.44; *p* < 0.0001) in addition to a decline in HVLT recall score at 6 and 12 months compared with the observation group (6, 44, 45).

QOL was also assessed in RTOG 0214 and showed that while global cognitive function and QOL was preserved between PCI and no PCI cohorts, there was decline in memory as measured by the HVLT in the group that received radiotherapy (6). Therefore, robust cognitive assessments may show neurocognitive decline in those receiving PCI, however, this does not always translate into patient's QOL being impacted.

There are currently efforts underway to try to deliver PCI in a way to try to mitigate cognitive effects. NRG Oncology CC003 “*Randomized Phase II/III Trial of Prophylactic Cranial Irradiation with or without Hippocampal Avoidance for Small Cell Lung Cancer”* is currently accruing patients in the hopes of enhancing the therapeutic ratio of PCI^1^; improve intracranial control while limiting neurocognitive toxicity. It has been hypothesized that radiation-induced injury to proliferating neuronal progenitor cells in the sub granular zone of the hippocampi may be responsible for the radiation induced NCF decline, thus, avoiding the hippocampal region of the brain may reduce cognitive side effects (46–48). The addition of neurocognitive protective agents is also being considered to further reduce the cognitive side effects of cranial irradiation (49).

## Systemic targeted or immunotherapies therapies for brain metastases prevention

An interesting approach to the treatment of brain metastases to try to mitigate the deleterious effect of radiotherapy to the brain has been to consider targeted or immunotherapies upfront to treat intracranial disease. The Chinese Thoracic Oncology Group conducted a randomized trial looking at patients with NSCLC with epidermal growth factor receptor (EGFR) mutations, who were naive to treatment with EGFR-tyrosine kinase inhibitors (TKI) or radiotherapy and had at least three metastatic brain lesions to either icotinib or WBRT (30 Gy in ten fractions of 3 Gy) plus concurrent or sequential chemotherapy for 4–6 cycles. In patients with EGFR-mutant NSCLC and multiple brain metastases, icotinib had significantly longer intracranial PFS than WBI plus chemotherapy. Therefore, icotinib might be a better first-line therapeutic option for this patient population (50).

In another recently published trial, 303 patients with untreated, advanced ALK-positive NSCLC were treated with alectinib (600 mg twice daily) or crizotinib (250 mg twice daily). The primary end point was PFS. Secondary end points were time to CNS progression, objective CNS response rate, and overall survival. A CNS response was appreciated in 17 of 21 patients in the alectinib group (CNS response rate, 81%; 95% CI, 58 to 95) and in 11 of 22 patients in the crizotinib group (CNS response rate, 50%; 95% CI, 28 to 72). Eight patients (38%) in the alectinib group had a CNS complete response (CR), compared to 1 patient (5%) in the crizotinib group. The median duration of intracranial response was 17.3 months in the alectinib group (95% CI, 14.8 to not estimable) and 5.5 months in the crizotinib group (95% CI, 2.1 to 17.3), respectively. A CNS response occurred in 38 of 64 patients in the alectinib group (CNS response rate, 59%; 95% CI, 46 to 71) and in 15 of 58 patients in the crizotinib group (CNS response rate, 26%; 95% CI, 15 to 39) in patients who had measurable disease. Twenty-nine patients (45%) in the alectinib group had a CNS CR, as compared with 5 patients (9%) in the crizotinib group. This was an important trial as it showed that in patients who harbor an ALK-mutation, targeted therapies can be effective in treating and *preventing* CNS progression (51).

Similar studies have also been performed in patients with metastatic melanoma. In a recently published trial, patients with asymptomatic melanoma brain metastases with no prior local CNS therapy were randomly assigned to cohort A (nivolumab plus ipilimumab, *n* = 36) or cohort B (nivolumab, *n* = 27). With a median follow up of 17 months (IQR 8–25), intracranial responses were achieved by 16 (46%; 95% CI 29–63) of 35 patients in cohort A and five (20%; 7–41) of 25 in cohort B. Intracranial CR occurred in six (17%) patients in cohort A and three (12%) in cohort B. The effectiveness of these therapies came at the cost of treatment-related adverse events which occurred in 34 (97%) of 35 patients in cohort A and 17 (68%) of 25 in cohort B. Grade 3 or 4 treatment-related adverse events occurred in 19 (54%) patients in cohort A and four (16%) in cohort B indicating that the combination therapy was more toxic (52).

Another EGFR-TKI Lapatinib has also shown effectiveness in the treatment of metastatic HER2 positive breast cancer to the brain based upon 2, Phase II clinical trials (53, 54). Addition studies have also shown that Lapatinib in combination with chemotherapy can decrease the rate of CNS relapse of Her2 positive disease from 6% down the 1–2%. Currently, the Radiation Therapy Oncology Group (RTOG) 1119 is evaluating the complete response rate in the brain at 12 weeks post WBRT based upon MRI with the addition of Lapatinib and WBRT compared to WBRT alone in women with Her2 positive disease that has metastasized to the brain^1^,^1^. Another agent that has shown activity in the treatment of HER2 positive metastatic breast cancer to the brain is neratinib. There are trials currently accruing to determine if neratinib combined with other systemic chemotherapies will show activity against CNS metastases (55).

An interesting concept based upon these promising results is whether systemic targeted or immunotherapies could be used in the prevention of disease as opposed to treatment of metastases that have already developed. Trial concepts are currently being generated at the cooperative group level to address this question.

## Conclusion

The prevention of metastases spreading to the CNS would have a significant benefit in preventing debilitating side effects. PCI has shown promise in preventing CNS metastases in ALL, LD and ED-SCLC, and NSCLC. However, a survival benefit has only been firmly established in ALL and SCLC. Some argue that in the absence of a survival benefit PCI should be omitted because of the neurologic and QOL sequelae that can occur in some patients. However, consideration needs to be given to the competing decline in cognition and QOL that can arise because of the development of CNS metastases. Novel radiation delivery techniques and targeted and immunotherapies may provide some hope of preventing CNS metastases without the negative impact on cognition and QOL.

## Author contributions

The author confirms being the sole contributor of this work and has approved it for publication.

### Conflict of interest statement

The author declares that the research was conducted in the absence of any commercial or financial relationships that could be construed as a potential conflict of interest.

## Footnotes

^1^https://www.rtog.org/ClinicalTrials/Welcome.aspx
